# Ovicidal, immunotoxic and endocrine disrupting effects of saponin on *Bulinus truncatus* snails with special emphasize on the oxidative stress parameters, genotoxicological, and histopathological alterations

**DOI:** 10.1007/s11356-023-27668-w

**Published:** 2023-06-05

**Authors:** Amina M. Ibrahim, Ali A. Al-Fanharawi, Hebat-Allah A. Dokmak

**Affiliations:** 1 Medical Malacology Department, Theodor Bilharz Research Institute, Imbaba, Giza, P.O:11635 Egypt; 2 Biology Department, College of Science, University of Al-Muthanna, Al-Muthanna, Iraq

**Keywords:** *B. truncatus*, Saponin, Biological, Immunotoxic, Biochemical alterations, Histopathology

## Abstract

*Bulinus truncatus* snail is one of the most medically important snails*.* The goal of this study was to evaluate the molluscicidal effect of saponin on these snails and study how it affects their biological functions. The present results showed that saponin had a molluscicidal activity against adult *B. truncatus* snails after 24h and 72h with LC_50_ (57.5 and 27.1 ppm, respectively) and had ovicidal acivity on the snails’ embryos. By studying the effect of the sublethal concentrations (LC_10_ 48.63 ppm or LC_25_ 52.83 ppm) exposure on *B. truncatus* snails, they resulted in significant decreases in the survivorship, egg-laying, and the reproductive rate compared to untreated snails. Both concentrations caused morphological changes to the snails’ hemocytes, where, after the exposure, granulocytes and hyalinocytes had irregular outer cell membrane and some cell formed pseudopodia. Granulocytes had large number of granules, vacuoles, while hyalinocytes’ nucleus was shrunken. Also, these concentrations resulted in significant increases in sex hormone levels (17β-estradiol and testosterone) in tissue homogenate of *B. truncatus* snails. It resulted in significant decrease in total antioxidant (TAO) activity, while, significantly increased lipid peroxidase (LPO) level, superoxide dismutase (SOD), nitrogen oxide (NO), and glutathione-S-transferase (GST) as compared to control group. Histopathological and genotoxicological damages occurred in snails’ tissue after exposure to these concentrations. Conclusion, saponin has a molluscicidal effect on *B. truncatus* snails and might be used for the control of *schistosomiasis haematobium.* Besides, these snails could be used as invertebrate models to reflect the toxic effects of saponin in the aquatic ecosystem.

**Graphical Abstract:**

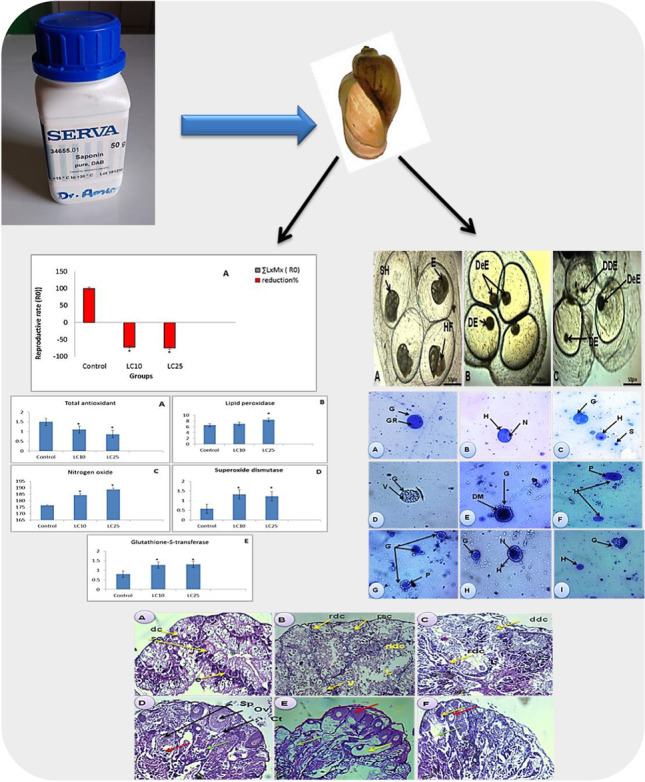

## Introduction

Schistosomatidae family, which includes the genus *Schistosoma*, required two hosts, intermediate and final host to complete their life cycle (WHO 2021; Morad et al. 2022). Some of these genera are responsible for cercarial dermatitis (Sodeman, 1989; Loker and Mkoji 2005) and many socioeconomic problems (WHO 2022). It was globally distributed in many parts of Africa, the Middle East, and south America (Ibrahim and Sayed 2021; Ibrahim et al. 2023).

The freshwater snail *Bulinus truncatus* (Audouin, 1827) (phylum Mollusca, class Gastropoda) is the intermediate host of *Schistosoma haematobium* and *S. bovis* (Xing et al. 2021; Hang et al. 2022) in Africa and the Middle East (Konan et al. 2022). Due to the ethics of scientific study, these snails could be employed as models to illustrate the impacts of specific materials in experimental studies to reduce the use of higher vertebrate models (Ibrahim and Sayed 2019; Ibrahim et al. 2021). Schistosomiasis control programs were performed by implementation of a national program that included chemotherapy and/or snail control either naturally or chemically (WHO 2020). The chemical control of these snails caused many deleterious consequences in the non-target organisms’ tissues that could be transferred to the higher food chain organisms like humans (Barathinivas et al. 2022). Besides, they are expensive and not degradable in the ecosystem (WHO 2014; Ibrahim et al., 2022).

Saponins are glucosides that are known to be a type of the plants’ secondary metabolites (Moses et al. 2014). It was extracted from *Quillaja saponaria* bark and it has been used as molluscicidal agent (Joshi et al. 2008; Adomaitis and Skujiene 2020). Saponins are promising choices for controlling schistosomiasis (Nair and Varalakshmi 2011; Ibrahim and Abdalla 2017; Augusto and de Mello-Silva 2018; Ibrahim and Sayed 2021). A recent study showed that saponin has molluscicidal activity against adult *B. alexandrina* snails with LC_50_ 70.05 mg/l and reasoned this toxic effect to the amphiphilic nature of saponin that resulted in the cell membrane disturbances (Ibrahim et al. 2023) and also, it has miracidicidal and cercaricidal activities on the free larval stages of *S. mansoni*. Another study showed that pure saponin is less toxic than the extracted plant parent by three to five times and this was done on the aquatic crustacean *Daphnia magna* and zebra fish (*Danio rerio*) embryos (Jiang et al. 2018). It also could be used as insecticidal, antimicrobial, antiviral, anticancer, and anti-inflammatory, antioxidant, and immunomodulatory (Asgharian and Ojani 2017; Trdá et al. 2019; Biswas and Dwivedi 2019). It can be used as bio- surfactants in *Daphnia galeata* and *Bosmina longirostris* ecotoxicological testing as it decreased the entrapment of these water fleas in the water surface film (Cui et al. 2017). Saponins could be used cautiously in the aquatic ecosystem because its higher concentration could exceed the water quality limit (Jiang et al. 2018).

Therefore, the aim of the present study is to investigate the toxicological impact of saponin on *B. truncatus* snails, then studying its impact on different biological, immunotoxicological, endocrine disruption, oxidative parameters alterations, and histopathological alterations of these snails after exposure.

## Materials and methods

### Saponin

Saponin was purchased from (SERVA Electrophoresis GmbH, D- 69115 Heidelberg- Carl- Benz- Str. 7) as white to beige pure powder extracted from *Quillaja saponaria* bark with 5% solubility in water. A stock solution was prepared by dissolving the powder in distilled water (on the basis of W/V), which was used to prepare the different serial concentrations.

### Snails rearing

*Bulinus truncatus* snails (5–8 mm) were obtained from the stock reared in Medical Malacology Department, Theodor Bilharz Research Institute (TBRI), Imbaba, Giza, Egypt. They were bred in plastic aquaria measured (16 × 23 × 9 cm), each aquaria with 10 snails/L in dechlorinated water (25 ± 1°C), pH: 7± 0.2, 12h/12h photoperiod was controlled and covered with glass plates. Optimization of the water hardness was done by using 30 mg/L calcium carbonate (CaCO_3_) (Eveland and Haseeb 2011). Blue-green algae (*Nostoc muscorum*), oven-dried lettuce leaves and Tetramin (*ad libitum*) were used for snail feeding. For collecting egg masses, small pieces 3×5 of polyethylene sheets were float in the aquaria (Pellegrino and Goncalves, 1965). Water was changed twice a week and dead snails were removed (Eveland and Haseeb 2011). Ethics for collecting and using snails have been done according to NENT (2019).

### Bioassay tests

A stock solution of 1000 ppm from saponin was prepared, and dilutions were performed for determining LC_10_, LC_25_, LC_50_, and LC_90_ against *B. truncatus* snails. Therefore, those snails were treated with concentrations of 45, 50, 55, 60, 65, and 70 mg/L of saponin for 24h or concentrations of 10, 15, 20, 25, 30 mg/l for 72h. Afterwards, snails were thoroughly washed and transferred to a clean dechlorinated water to recover for 24h. For each concentration, three replicates were used, each of which 10 snails (5–8mm)/L. Mortality of snails was recorded (WHO 1983) and analyzed to obtain the lethal concentration values by probit analysis software (WHO 1965). For control groups, dechlorinated water was allowed to run along with the test samples with tri-replicate manner. About 210 snails were used in this experiment.

### Effect of saponin on the survival and the reproductive rates of adult snails

One hundred and eighty *B. truncatus* snails (6–8mm) were exposed for 24 h/day for 2 weeks to the concentrations LC_10_ or LC_25_ of saponin. Three replicates, each of 10 snails/L, were prepared for each concentration, another group considered as control group was maintained in dechlorinated water, the following parameters were weekly recorded: Lx (the survival rate as a proportion of the correct one at time of exposure in weeks (x), fecundity (Mx) (the number of eggs/snail/week), and *R*_0_ (the reproductive rate which is the summation of LxMx during the experimental period) (El-Gindy and Radhawy, 1965).

### Investigation of ovicidal activity

Egg masses on the sheets were transferred to petri dishes, where they were exposed to the tested concentrations. For each concentration, 100 eggs were used and assays were repeated three times. At the end of exposure (24 h), egg masses were transferred to petri dishes with dechlorinated water and were examined daily under a stereomicroscope up to the 7th day (Ibrahim and Abdalla 2017).

### Investigation of the hemocytes morphological alterations after exposure to the sublethal concentration

Hemolymph samples were collected from each group of tested snails that was exposed to LC_10_, LC_25_, and control snails group by removing a small portion of the shell and inserting a capillary tube into the heart (Nduku and Harrison 1980). Hemocytes monolayers were prepared by placing 10 μl of hemolymph on a glass slide and were allowed to be adhered the glass surface for 15 min at room temperature. Hemocytes were fixed with absolute methanol for 5 min and then stained with 10% Giemsa stain (Aldrich) for 20 min (Abdul-Salam and Michelson 1980), then examined under microscope.

### Assay for the biochemical alterations

#### Tissue preparation

From each exposed and the control groups, ten snails were crushed between two slides, and their soft tissues were withdrawn, weighed (1g tissue/10 ml phosphate buffer) and then homogenized by a glass Dounce homogenizer. The homogenates were centrifuged at 3000 rpm for 10 min and the supernatants were used.

##### Investigation of steroid sex hormones (testosterone and 17β-estradiol) after exposure to saponin

The steroid hormones (testosterone, 17β-estradiol) were determined in tissue of snails survived post exposure to LC_10_ or LC_25_ of saponin and in the control group. Hormone concentrations (T and E) were assayed for all groups according to the manufacturer instructions of T EIA kit (Enzo Life Science, Michigan, USA, ADI-900-065) and E EIA kit (Cayman Chemical Page 6/18 Company, Michigan, USA, item no. 582251). These kits were used for the quantitative determination of testosterone and estradiol in a colorimetric competitive enzyme immunoassay with results in 3 h.

##### Investigation of the antioxidant biomarkers (SOD, GST, MDA, and TAC)

Biodiagnostic kits (Biodiagnostic Dokki, Giza, Egypt) were used for the determination of SOD and it relied on the ability of the enzyme to inhibit the phenazine methosulphate-mediated reduction of nitroblue tetrazolium dye (Damerval et al. 1986). Also, glutathione S transferase (GST) kit is a colorimetric assay designed for the quantification and detection of glutathione in tissue samples and measured according to (Beutler 1963). In addition, tissue malondialdhyde (lipid peroxide) was done colorimetrically according to Ohkawa et al. (1979) and total antioxidant capacity was estimated by kit (Cat. No. TA 2513) and based on the reaction of antioxidants in the sample with a defined amount of exogenously provide hydrogen peroxide (H_2_O_2_). The antioxidants in the sample eliminate a certain amount of the provided hydrogen peroxide and the residual hydrogen peroxide is determined colorimetrically by an enzymatic reaction (Koracevic et al. 2001).

### Investigation of the histopathological alterations

The snails were treated with LC_10_ and LC_25_ of saponin as mentioned before. Control groups were simultaneously carried out. Three replicates (10 snails/ L for each) were used for both control and the tested groups. Thereafter, the snail’s hermaphrodite gland was dissected out of their shells and was fixed using Bouin's fixative, then embedded in wax blocks, sectioned (5–8μm), and stained with hematoxylin and eosin (Mohamed and Saad 1990). Similarly, sections of control snails’ hermaphrodite glands were prepared.

### Molecular changes by comet assay

Snails (8–10 mm) were subjected to LC_10_ or LC_25_ of saponin for 24h and then DNA damage was measured by single cell gel assay according to Singh et al. (1988) and Grazeffe et al. (2008).

### Statistical analysis

Statistical analyses were run on IBM compatible PC using SPSS for windows statistical package (SPSS, 2006). Lethal concentrations were calculated using Probit analysis software (Finney 1971). The mortality rates of the experimental groups were compared using Pearson’s Chi-square test. Values of the biochemical parameters were expressed as mean± SD. Student’s *t*-test was applied to locate significant changes between control and treated groups (Murray 1981).

## Results

The tested saponin in the current study revealed a toxic molluscicidal activity against the intermediate host of *S. haematobium* after a 24-h and 72-h exposure with different concentrations. The mortality of *B. truncatus* snails was positively concentrations dependent. The LC_50_ value was 57.5 and 27.1 ppm after 24-h and 72-h exposure, respectively. Interestingly, slope value indicated that the lethal concentration probability lines (LCP) of saponin was steep, which indicated that the toxic effect of saponin is concentration dependent (Table 1).

**Table 1:** Molluscicidal activity of saponin against *Bulinus truncatus* snails (24 and 72 h of exposure)

Saponin	LC_10_(ppm)	LC_25_(ppm)	LC_50_(ppm)	Confidence limits of LC_50_ (ppm)	LC_90_(ppm)	Slope
24 h	48.63	52.83	57.50	(52.64–62.35)	66.36	1.12
**72 h**	17.3	22.01	27.1	(19.22–32.37)	36.89	1.1

**Fig. 1:**
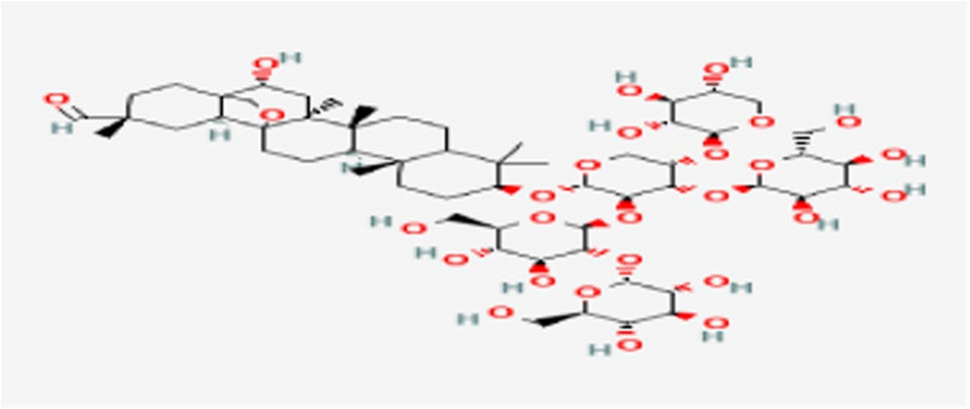
Structure of saponin

Results revealed that the biological parameters that are tested in *B. truncatus* snails exposed to either LC_10_ 48.63 ppm or LC_25_ 52.83 ppm of saponin recorded decrease values in the survivorship, egg-laying, and the reproductive rate (*R*_0_) of snails compared to untreated snails (Table 2; Fig. 1).

**Table 2:** Some biological parameters, survivorship (Lx), fecundity (Mx), and reproductive rate (*R*_0_) of *Bulinus truncatus* snails exposed to synthetic saponin

Observation time (week)	Control			LC_10_ (ppm)			LC_25_ (ppm)		
	LX	MX	LXMX	LX	MX	LXMX	LX	MX	LXMX
0	1.0	1.2	1.2	1.0	1.2	1.2	1.0	1.2	1.2
1	1.0	2.90	2.90	0.8	1.1	0.88	0.6	0.77	0.4
2	0.9	1.77	1.59	0.6	0.0	0.0	0.5	0.0	0.0
3	0.9	1.1	0.99	0.4	1.1	0.44	0.3	1.0	0.3
4	0.8	0.0	0.0	0.2	0.0	0.0	1.0	0.0	0.0
5	0.8	1.1	0.88	0.1	0.0	0.0	0.0	0.0	0.0
*R*_0_± S.D			7.56±0.03			2.52±0.11*			1.9±0.08*
Reduction % of *R*_0_						71.80			74.86

^*^Significant at *P*<0.05

**Fig. 2:**
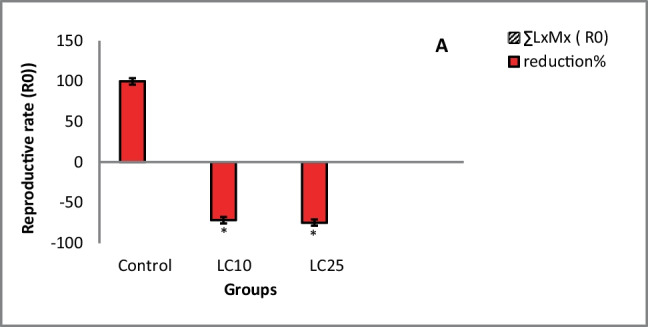
Reduction (mean % ± S.D) of reproductive rate (R_0_) of *Bulinus truncatus* snails exposed to saponin

The present results showed that *B. truncatus* ova exposed to either LC_10_ 48.63 ppm or LC_25_ 52.83 ppm of saponin showed delayed developmental stage of embryos, dead and degenerated dead embryos (Fig. 2).

**Fig. 3:**
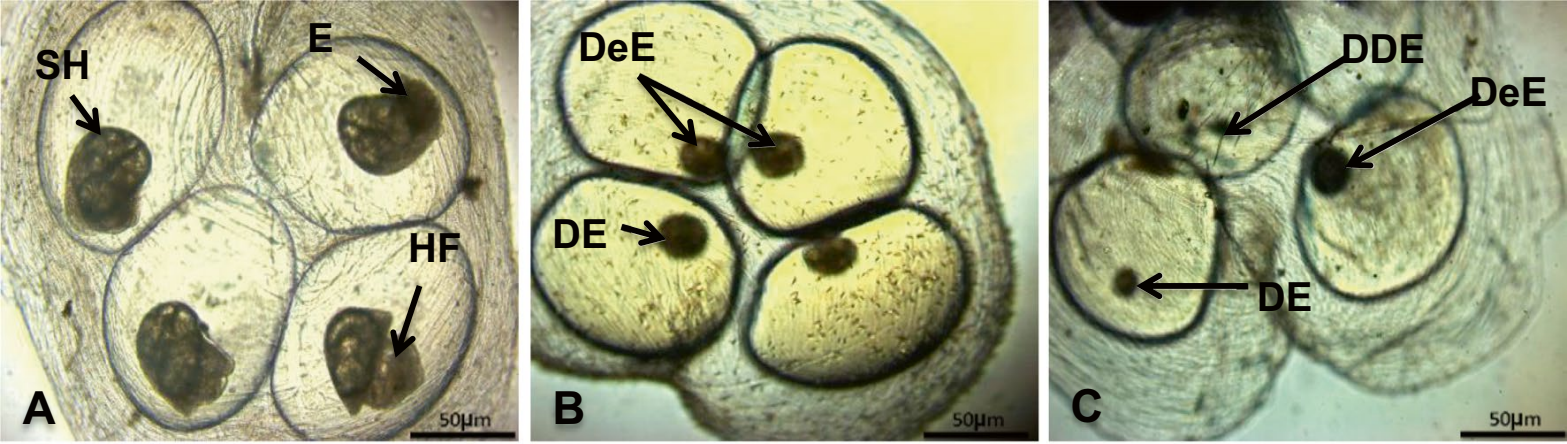
*B. truncatus* ova exposed to LC_10_ and LC_25_ concentration of saponin (after 1 week). **A** Normal healthy embryo with eyes (E) on the head foot (HF) and with shell (SH). **B** Developmental delayed embryo (DeE) and dead embryo (DE) after exposure to LC_10_ of saponin; **C** degenerated dead embryo (DDE), dead embryo (DE), and delayed embryo (DeE) after exposure to LC_25_ of saponin (50× magnification)

**Fig. 4:**
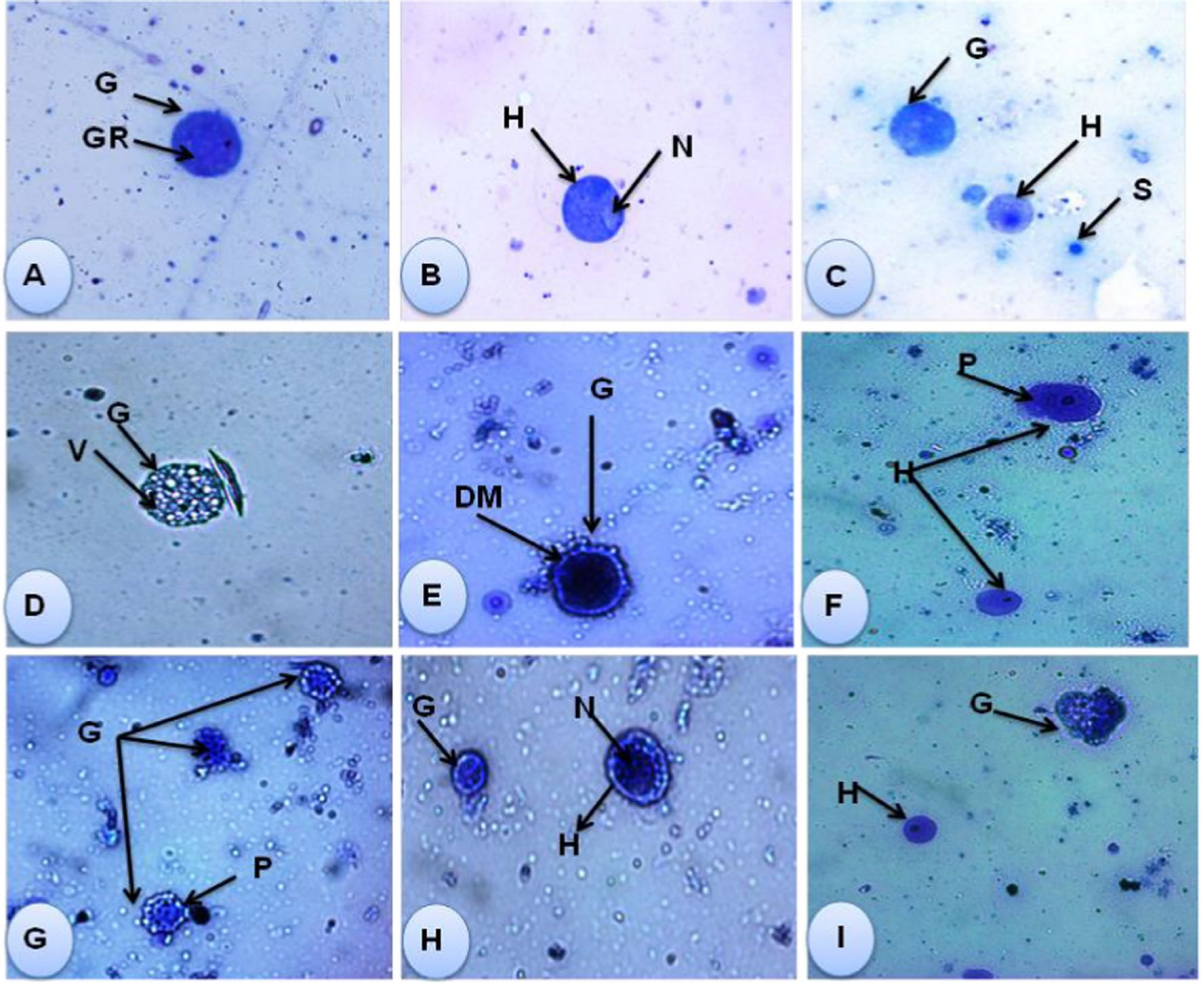
Photomicrographs show *B. truncatus* hemocytes (×40). (1) Normal hemocytes (**A**, **B**, **C**). (2) After exposure to LC_10_ 48.63 ppm of saponin (**D**, **E**, **F**). (3) After exposure to LC_25_ 52.83 ppm of saponin (**G**, **H**, **I**). CY, Cytoplasm; DM, double membrane; PS, pseudopodia; G, granulocyte; GR, granules; H, hyalinocyte; N, nucleus; S, round small; V, vacuoles

The current investigation elucidated the presence of two types of large cells (granulocytes and hyalinocytes) and one small type of cells (Fig. 3, A, B, and C). Exposure of *B. truncatus* snails to either LC_10_ 48.63 ppm or LC_25_ 52.83 ppm of saponin caused morphological changes to these cells. Where, after the exposure, granulocytes had plenty of granules, vacuoles, irregular cell membrane, and forming pseudopodia. The nucleus of some hyalinocytes was shrinked, some had irregular outer cell membrane and some cell formed pseudopodia (Fig. 4, D, E, F, G, H, and I).

**Fig. 5:**
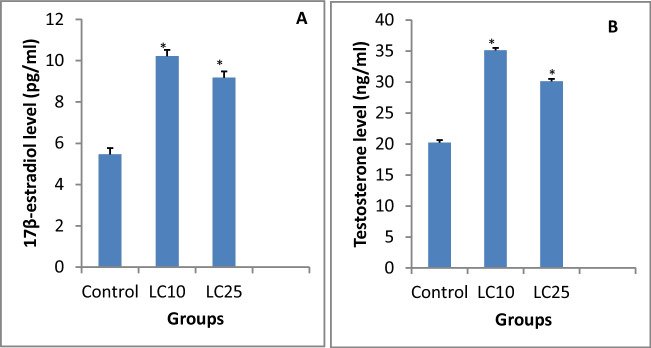
Effect of saponin (LC_10_ and LC_25_ concentration) on: **A**-17β-estradiol and **B** testosterone in tissue homogenate of *Bulinus truncatus* snails

Changes in sex hormone levels (17β-estradiol and testosterone) in tissue homogenate of *B. truncatus* snails exposed to saponin were occured. After exposure to LC_10_ 48.63 ppm or LC_25_ 52.83 ppm of saponin both the level of 17β-estradiol and testosterone hormones were significantly increased (*p*< 0.05) compared to control group (Fig. 5).

**Fig. 6:**
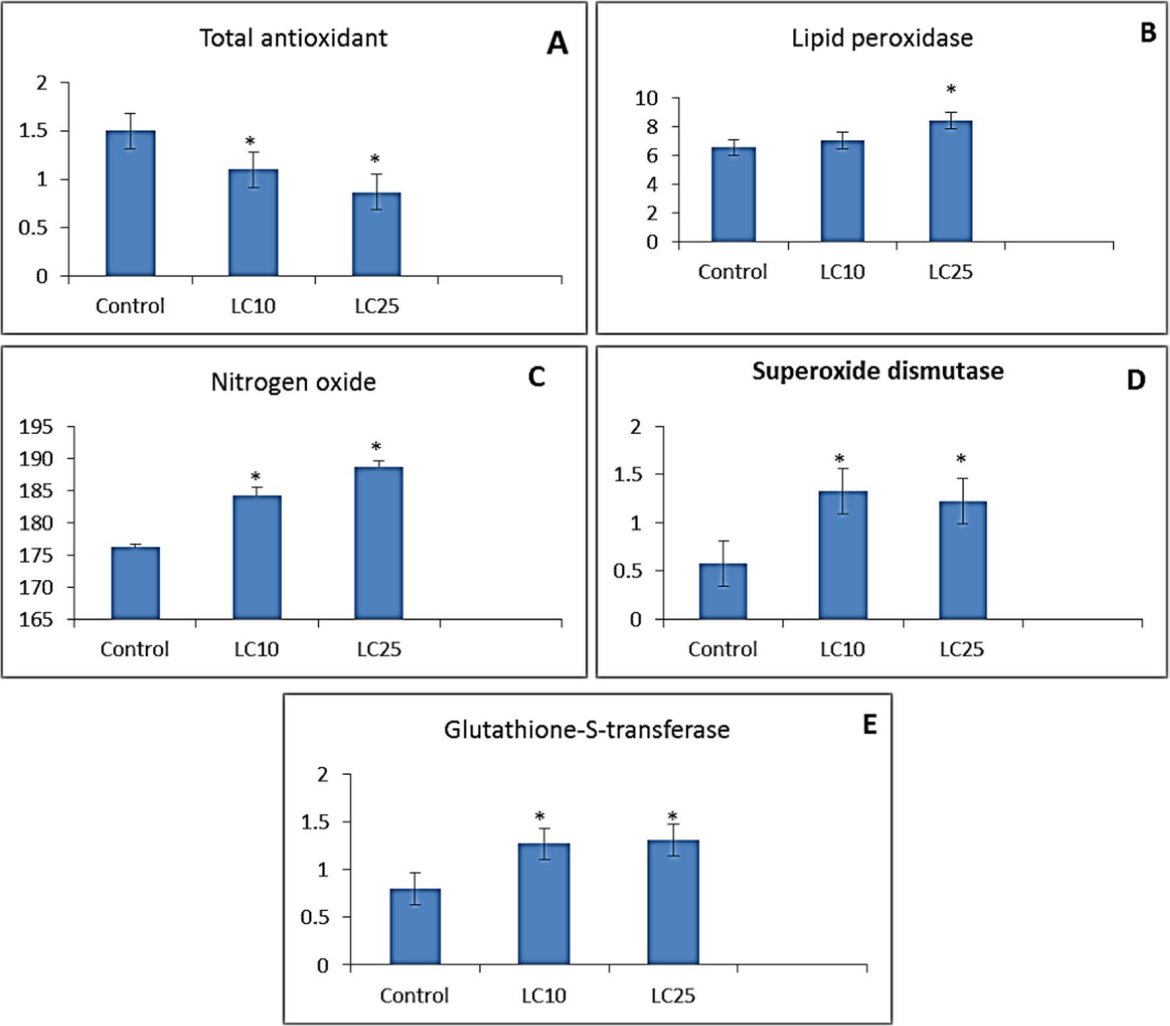
Effect of saponin (LC_10_ and LC_25_ concentration) on **A** total antioxidant; **B** lipid peroxidase; **C** nitrogen oxide; **D** superoxide dismutase; and **E** glutathione-s-transferase in tissue homogenate of *Bulinus truncatus* snails

The present data indicated that exposure of *B. truncatus* snails to either LC_10_ 48.63 ppm or LC_25_ 52.83 ppm of saponin significantly decreased TAO activity; while, they significantly increased lipideperoxidase (LPO) level, superoxidedismutase (SOD), nitrogen oxide (NO), and glutathione-s-transferees (GST) as compared to control group (Fig. 6).

**Fig. 7:**
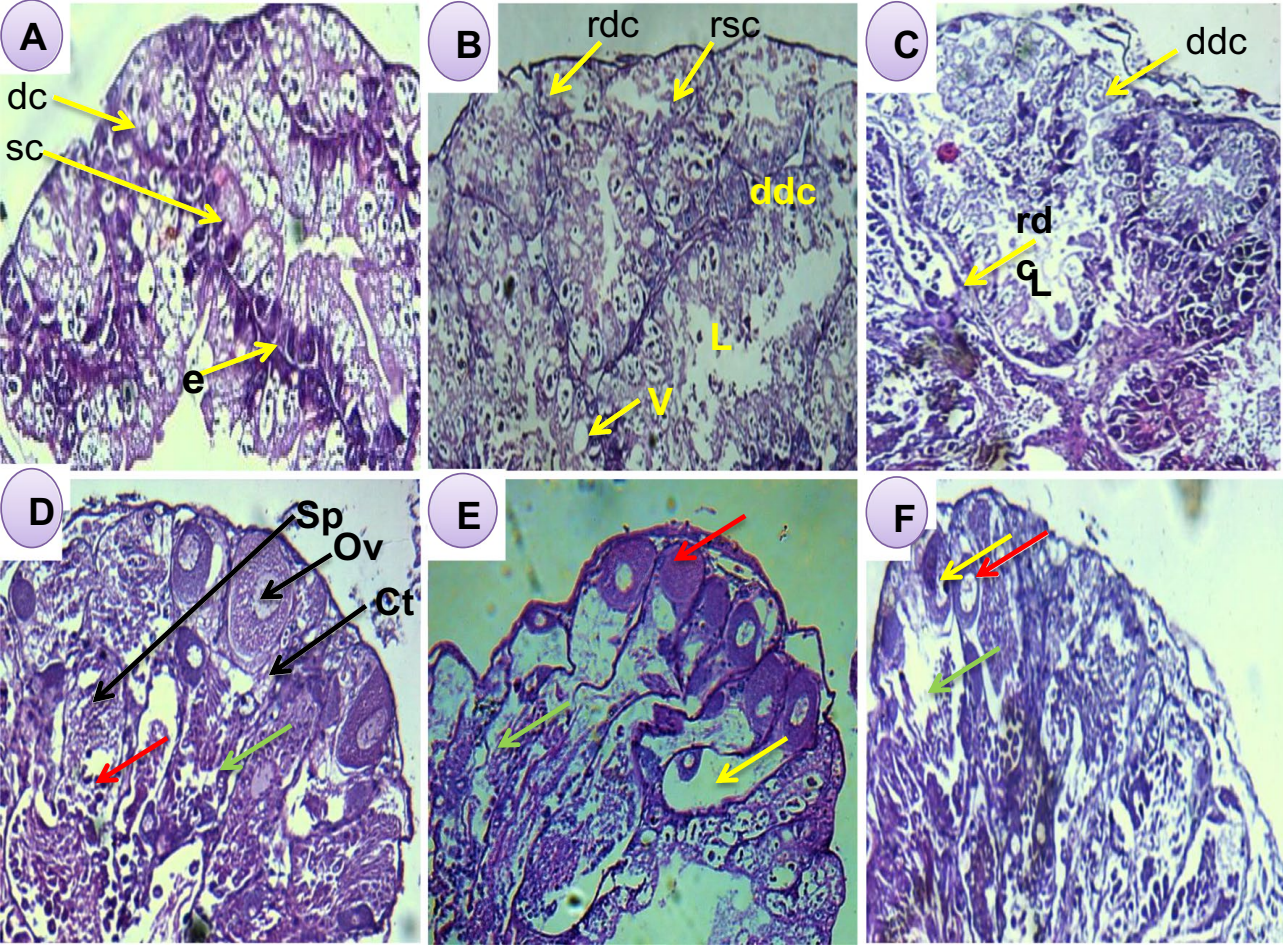
**A** Light photomicrograph of the digestive gland transverse section of normal *Bulinus truncatus* snails (hematoxylin and eosin stain ×40) with normal digestive cells (dc),normal secretory cells (sc), lumen (l), and epithelial layer (e). **B** Snails exposed to LC_10_ of saponin and **C** treated with LC_25_ of saponin showing: ruptured digestive cells (rdc), shranking, swelling, vacuole (V) formation and degenerated digestive cells (ddc). **D** Light photomicrograph of transverse section in the hermaphrodite gland of *Bulinus truncatus* (control), showing: Ov, mature ova; Sp, developed sperms; Ct, connective tissue, spermatocyte (red arrow) and oocytes (green arrow); **E** treated LC_10_ and **F** treated LC_25_ of saponin: showing degeneration ova (red arrow), scattered degenerated sperm (green arrow) and vacuolated ova (yellow arrow)

Examination of the histological sections of the digestive and hermaphrodite glands transverse of snails treated with LC_10_ and LC_25_ of saponin (Fig. 7 A, B, and C) showed cleared rupture and swelling of the digestive and secretory cells compared to control snails. Moreover, the hermaphrodite gland transverse sections of snails treated with the same concentrations of saponin (Fig. 7 D, E, and F) showed varying degrees of degenerations, atrophy and rupture of different cell types, ova and sperms in the glands’ acini. Furthermore, the most prominent damages were clear for several vacuolated ova, degeneration and scattered sperms after exposure to LC_25_ of saponin when compared to the control group.

**Fig. 8:**
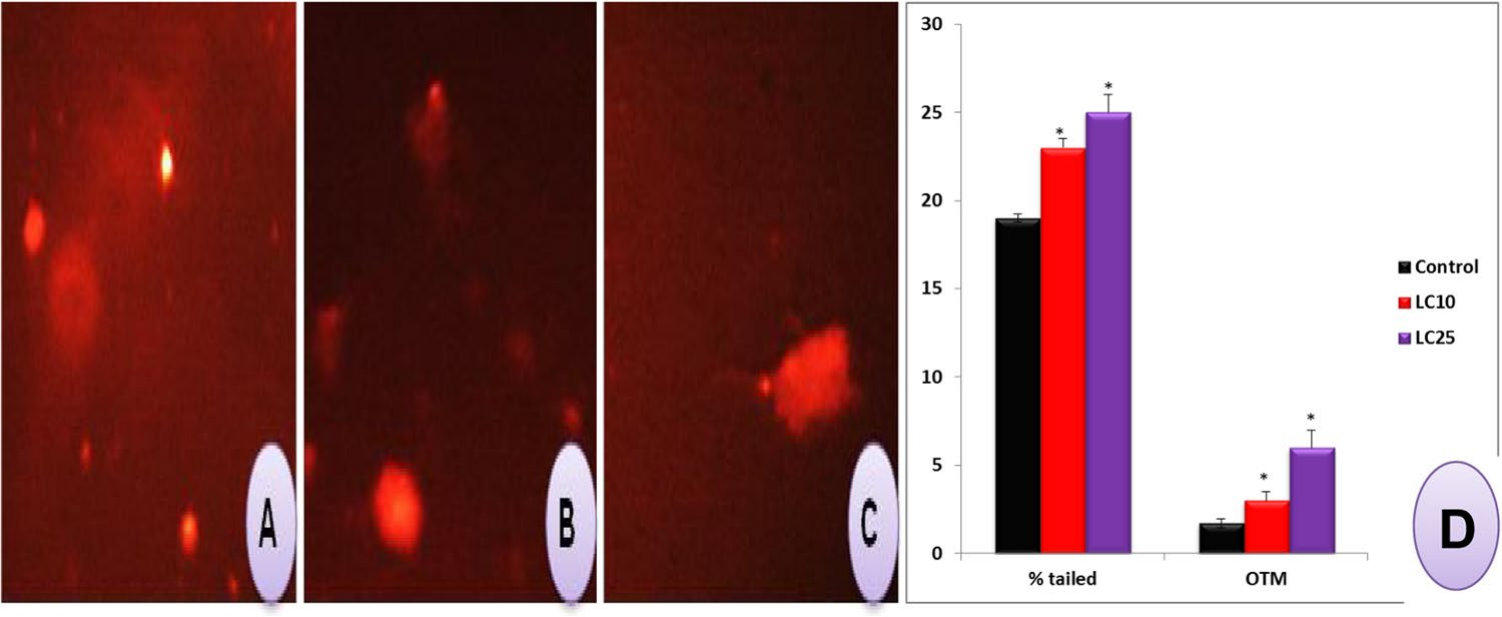
Shows results of the alkaline comet assay according to the extent of DNA migration. **A** Control; **B** LC_10_; **C** LC_25_ of saponin. **D** Histogram shows the % tailed and olive tail moment (OTM) of exposed snails to LC_10_ and LC_25_ of saponin

The results of the alkaline comet assay showed that the percent tailed comet and the olive tail moment (OTM) of snails exposed to the sublethal concentrations of saponin were increased significantly (*p*< 0.05) than the control snails (Fig. 8).

## Discussion

World Health Organization has tested thousands of agent compounds for the eradication of snails and reported that molluscicides of plant origin were more promising as they could not pollute the environment and were biodegradable (WHO 1990). The present results indicated that saponin induced toxic effect to *B. truncatus* snails a study that agrees with the data of (Zheng et al. 2021) which recorded the toxicity effects of novel triterpene saponins against *B. glabrata* snails. This could be due to that saponin has specific ability to perforate the cell membranes (Lorent et al. 2014). Also, the molluscicidal activity of Saponins might be due to their detergent effects on the soft body of these snails (Bahgat et al. 2018).

Results showed that after exposure of *B. truncatus* snails to either LC_10_ or LC_25_ of saponin, the survivorship, egg-laying, and the reproductive rate were significantly decreased compared to untreated snails. These alterations might be due to that *B. truncatus* snails had no operculum and so, their soft bodies were continuously in contact with the tested saponins during the experiment (He et al. 2017). Similar observations were noticed on the freshwater snail *Lanistes lybicus* tissues after exposure to saponins in a dose-dependent way (Akinpelu 2012). Moreover, the survival rate of *Biomphalaria pfeifferi*, and *Bulinus globosus* snails were decreased after exposure to *Cucurbita maxima* seed extracts concentration (Mtemeli et al. 2021). Molluscicides could induce death by disrupting the physiological processes that could explain the high mortality rates and long periods of ceasing egg-laying during the recovery period (McCullough et al. 1980). Also, these reductions in Mx and *R*_0_ of *B. truncatus* post-saponin exposure might be due to the harmful effects on the regulation of the oviposition. In addition to the interruption of their physiological activities, confirmed by the deteriorations on the levels of the antioxidant enzymes in the snails’ tissues. This mirrored on the developmental stages of the embryos, where the present results showed that *B. truncatus* ova exposed to either LC_10_ or LC_25_ of saponin showed delayed, dead, and degenerated dead embryos. These alterations were caused because of the great surface area for contact that allowing for the penetration of saponin (Li et al. 2014). Araújo et al. (2018) reasoned the embryotoxicity of saponin to its absorption through the cellular membrane causing changes in its permeability (Li et al. 2009).

Snails immune system consists of cellular and humoral components (Le Clec’h et al. 2016) (Baroudi et al. 2020). Hemocytes have three morphological shapes (Larson et al. 2014; Fried 2016), undifferentiated small, hyalinocytes, and granulocytes (Ibrahim et al. 2018). The immunological responses in *B. alexandrina* snails have been used as biomarkers of exposure to environmental stressors (Ibrahim and Sayed 2020). The current investigation elucidated the presence of two types of large cells (granulocytes and hyalinocytes) and one small type of cells. Exposure of *B. truncatus* snails to either LC_10_ or LC_25_ of saponin resulted in deformation of cell shape where, both granulocytes and hyalinocytes had irregular cell membrane, vacuoles, and forming pseudopodia, while granulocytes had plenty of granules. The nucleus of some hyalinocyte was shrinked. The pseudopodia formation by cells is an immunological action of these cells to get rid of the foreign materials (Guria 2018; Ibrahim et al. 2022a). Similarly, Morad et al. (2022) reported after exposure to LC_10_ (31.82 mg/l) of selenium nanoparticles, the granulocytes had many granules and pseudopodia and the hyalinocytes had incomplete cell division with irregular cell membrane and forming pseudopodia. In a good accordance with this study, Ibrahim et al. (2019) elucidated that granulocytes had many granules and pseudopodia, while hyalinocytes had a shrunken nucleus after exposure of *B. alexandrina* to pendimethalin, butralin, or glyphosate isopropyl ammonium herbicides.

Testosterone and 17β-estradiol hormone required in the gonadal development and might be used as a biomarker for molluscicides toxicity (Omran and Salama 2016). The present results showed that both the level of 17β-estradiol and testosterone hormones were significantly increased after the exposure of *B. truncatus* snails to LC_10_ or LC_25_ of saponin compared to control group. Swan et al. (2000) reported that saponin could interfere with the endocrine functions leading to the increase in sex hormones levels.

These findings were in a good accordance with Morad et al. (2022) who reported elevated levels of both testosterone and 17β-estradiol after exposure of *B. alexandrina* snails to the sublethal concentration with SeNPs Myco-Synthesis. These alterations were due to the androgenic stimulatory action of saponin which resulted in the increase in sex hormones (Dasofunjo et al. 2020).

Antioxidant enzymes were used as a biomarker for stressors in both marine and freshwater organisms (Tellez-Bañuelos et al. 2009). The aquatic stressors might result in reproductive, oxidative stress parameters and teratogenic alterations in the aquatic organisms (Pašková et al. 2011). The alterations in SOD, CAT, GST, GR, and MDA levels could be useful biomarkers of aquatic system pollution (Khalil et al. 2017). The present data indicated that exposure of *B. truncatus* snails to either LC_10_ or LC_25_ of saponin significantly decreased TAO activity, while, increased LPO level, SOD, NO, and GST as compared to control group. The reduction in total antioxidant level in tissues of *B. truncatus* snails exposed to saponin due to the severe oxidative stress and reactive oxygen production (ROS) which could lead to suppression of this enzyme and alterations in other parameters (Xu et al. 2009). The oxidative stress might be the main cause of delaying the sexual maturation and puberty onset (Pizzino et al. 2017). Also, the alterations in LPO level, SOD, NO, and GST levels might due to the effect of saponin on membrane fluidity (Halliwell and Gutteridge 2015) and might cause damages to DNA, lipids, and proteins (Di Giulio and Meyer 2008). The antioxidant enzymes played an important role in the elimination of ROS and modulation of the living organisms’ response to the oxidative stressors (Pizzino et al. 2017). Nitric oxide (NO) is one of the innate immune defense mechanisms which lead to kill the invading pathogens (Ray et al. 2013; Ibrahim and Hussein 2022). Wang et al. (2018) reported increase level of NO of *B. straminea* snail after exposure to pyridyl phenyl urea derivatives.

Examination of the histological sections of the digestive glands of snails treated with LC_10_ and LC_25_ of saponin showed scattered vacuole, rupture and swelling of the digestive and secretory cells compared to control snails. Moreover, the hermaphrodite gland sections of snails treated with the same concentrations of saponin showed degenerations, atrophy and rupture of different cell types, ova and sperms in the glands’ acini. These could be due to the genital organs suffered from the saponin treatments which could resulted to decrease their oviposition for some weeks and these may explain why that the reproduction rate (*R*_0_) of exposed snails was reduced. In these regards, Abdel-Tawab et al. (2022) reported great damages in both hermaphrodite and digestive glands represented mainly as degeneration rupture and vacuolation of the digestive, secretory cells, sperms, and ova after exposure to cerium oxide nanoparticle synthesized with *Moringa oleifera* seeds at concentration 314.5 mg/l. In 2019, Ibrahim and Bakry (2019) stated that chlorophyllin extracted from deep-frozen leaves of *M. oleifera* plant exerted deleterious effects in the digestive gland of *B*. *alexandrina* snails treated with LC_25_ of water soluble chlorophyllin, represented by deformation of secretory cells, disintegration of the digestive cells, and rupturing the connective tissue between the gland tubules. Moreover, Dokmak et al. (2021) reported that destruction of the hermaphrodite and digestive glands cells of *B*. *truncatus* snails treated with cupper chlorophyllin and magnesium chlorophyllin and reasoned these damages to the harmful effects exerted by such agents during the photosensitization process.

DNA damages might be used as biomarkers of aquatic pollution in snails (Abdel-Tawab et al. 2022). Alkaline comet assay is a simple, fast, and reliable technique that was used to detect DNA single-strand breaks (Ibrahim et al. 2018). The present investigation showed that both the % of tailed cells and the olive tail moment (OTM) of snails exposed to the sublethal concentrations of saponin were increased compared with the control snails. Similarly, Ibrahim et al. (2023) stated that exposure of *B. alexandrina* snails to LC_10_ or LC_25_ concentrations of saponin caused genotoxic effects and downregulated the metabolic cycles for both genes (cytochrome oxidase subunit I (COI) and NADH dehydrogenase subunit 1 (ND1). These effects might be due to the oxidation of DNA bases resulting in strand breaks (Caixeta et al. 2020) after the exposure to DNA damaging materials compared to the control group (Ye et al. 2012).

## Conclusion

Therefore, saponins could be used as a molluscicide against the intermediate host of schistosomiasis and hence decrease the spread of this disease. Also, *B*. *truncatus* snails could be used as bio-indicators to reflect the effect of saponin on the biological system of organisms.

## Data Availability

Data are available on request from the authors
